# Systematic review of stranger homicides by psychotic individuals

**DOI:** 10.1093/schbul/sbaf246

**Published:** 2026-03-21

**Authors:** Jenna Räsänen, Ilkka Ojansuu, Jari Tiihonen, Johannes Lieslehto, Markku Lähteenvuo

**Affiliations:** Department of Forensic Psychiatry, University of Eastern Finland Niuvanniemi Hospital, Kuopio, Finland; Department of Forensic Psychiatry, University of Eastern Finland Niuvanniemi Hospital, Kuopio, Finland; Department of Forensic Psychiatry, University of Eastern Finland Niuvanniemi Hospital, Kuopio, Finland; Department of Clinical Neuroscience, Karolinska Institutet, Stockholm, Sweden; Center for Psychiatry Research Stockholm City Council,, Stockholm, Sweden; Department of Forensic Psychiatry, University of Eastern Finland Niuvanniemi Hospital, Kuopio, Finland; Department of Clinical Neuroscience, Karolinska Institutet, Stockholm, Sweden; Institute for Molecular Medicine Finland, University of Helsinki, Helsinki, Finland; Department of Forensic Psychiatry, University of Eastern Finland Niuvanniemi Hospital, Kuopio, Finland

**Keywords:** psychosis, violence, schizophrenia

## Abstract

**Background and Hypothesis:**

Individuals with psychosis have an increased risk of committing and being victims of violence. There are frequent media reports of psychotic individuals assaulting strangers, which may cause fear and stigmatization among the general public. We hypothesize that homicides targeting strangers by psychotic individuals are rare.

**Study Design:**

Systematic review and meta-analysis to assess what percentage of homicide offenders suffering from psychosis target strangers. Medline database was searched with search term ‘psychosis OR schizophrenia AND homicide’ from inception to 10/2024. Articles published in peer-reviewed journals, written in English, and reporting the total number of homicide offenders with psychosis (ICD-10: F20, F22, F25, F30-F31, F32.3, F29) and stranger victims were included. PRISMA guidelines were followed. Studies with inadequate data were excluded. A random-effects meta-analysis using meta and metafor packages in R version 4.4.2 was conducted using the restricted maximum-likelihood (REML) method to account for variability across studies. The primary outcome was the pooled rate of stranger homicides among individuals with psychosis who committed a homicide, expressed as a proportion with 95% confidence intervals (CIs).

**Study Results:**

Thirteen studies were included, comprising a total of 1,438 perpetrators who had killed 177 strangers. Meta-analysis of these studies indicates that 12.7% (95% CI: 7.85–17.56 and heterogeneity I^2^ 89.49%) of the homicides by psychotic individuals are targeted at strangers. Male gender explained 26.9% of between-study variance (P-value<0.05).

**Conclusions:**

Although still rare, the percentage of homicides committed by psychotic individuals and targeted at strangers is higher than previously reported.

## Introduction

The lifetime prevalence of any psychotic disorder has been estimated to be 2,6 - 4,2 %^1-3^ and below 0.7 % for schizophrenia.^4^^,^^5^ Psychotic disorders have been associated with an increased risk of committing violent acts or homicide, but estimates of the increase in risk vary widely depending on how comorbid substance use disorders are taken into account.^6-8^ Around 6.6 % of all homicide offenders suffer from schizophrenia indicating that the prevalence of schizophrenia is higher among homicide offenders than in the general population.^9^^,^^10^ About 82 % of the crimes committed by people with a psychotic disorder have been thought to be explained by the symptoms of their disorder, especially delusions, which have been reported to account for up to 46 % of the crimes.^7^ However, as comorbid substance use disorders and personality disorders also markedly increase the risk of violence and homicide, but might not always directly be linked to symptoms of psychosis, the interplay between these comorbidities and psychotic symptoms in increasing the risk of violent behavior is still somewhat obscure.^6^^,^^11^^,^^12^ It has, however, been postulated, that the motives and situational factors behind violence and homicides committed by psychotic and non-psychotic offenders might be different.^13^ One difference between the homicides committed by psychotic and non-psychotic offenders has been target selection.^13^ Psychotic individuals have been shown to target violence towards their families, although different victim profiles have been shown for different psychotic disorders, such as patients with schizophrenia being more likely to kill a stranger than patients with delusional disorder.^14^ A study of stranger homicide in England and Wales in 1996-1999 found that individuals with schizophrenia committed 7,8 % of all homicides and 4,3 % of all homicides targeted at strangers during that time period.^15^ A meta-analysis of the prevalence of homicidal acts targeted at strangers by psychotic individuals was conducted in 2009, reporting these to be rare, as only about 9.0 % of the homicides were targeted at strangers.^16^ The meta-analysis included seven studies between the years 1955-2004 from five different countries, and included both homicides and attempted homicides. The sex/gender of the offender also seems to affect target selection. In England and Wales, the proportion of homicides targeted at strangers was 35 % for male offenders in 2018 and 22 % in 2017, whereas for female offenders it was 17 % in 2018 and 8,5 % in 2017.^17^ For Finland, the rates were 11 % for males and 3,0 % for females in 2017 and 16 % for males and 2,7 % for females in 2016.^18^ For Australia, combined rates for both sexes were 11 % in 2017-2018^19^ and 16% in 2016-2017.^20^ In the United States the rates were 9,7 % for both sexes in 2017^21^ and 26 % for males and 12 % for females during the years 1980-2008.^22^ For Russia the rates were 35 % for males and 26 % for females in 1981-1998.^23^ In summary, homicides by females are significantly less frequent in general and less frequently targeted toward strangers.

In the current article, we expand the previous meta-analysis with additional studies from different countries and years. This work is relevant as the prevalence of homicides varies between countries and years,^24-27^ and so may target selection. Homicides committed by individuals with psychotic disorders and targeted at strangers attract significant public and media attention. Such cases often shape societal perceptions of mental illness and can contribute to enduring stigma. From a forensic psychiatric standpoint, identifying the specific circumstances and profiles associated with these rare but highly salient events may inform targeted risk assessment, service planning, and preventive interventions. Furthermore, quantifying and contextualizing the risk of stranger homicide has implications for legislation and public policy, as it supports the development of proportionate and effective measures for both public protection and the rights of individuals with mental disorders. We set out to perform an updated meta-analysis on the risk of strangers being victims of homicides committed by psychotic individuals.

## Materials and Methods

### Material Review and Inclusion/Exclusion

We searched the Medline database for studies using the PubMed search engine with search terms “psychosis OR schizophrenia AND homicide” in 10/2024. The search was not restricted by publication year and yielded a total of 1103 articles. From this article set, we included articles published in peer-reviewed journals, written in English, and which reported the total number of homicide offenders and stranger victims. We further reduced the articles to those with participants diagnosed with a psychotic disorder, such as schizophrenia (ICD-10: F20, F22, F25), affective psychoses (F30, F31, F32.3) or other psychotic disorder (F29). Studies without diagnostic information, without original data, with participants under 15 years of age or inadequate data on the criminal offense (completed or attempted homicide) were excluded. We also excluded studies that included only offenders convicted of specific types of homicides, such as those involving thrusting individuals onto train/metro tracks, which might have skewed results. We assumed that the terms “homicide” and “murder” referred to completed homicides and excluded other crime descriptions, such as “attempted murder/homicide”. The records were screened for inclusion or exclusion by at least two of three independent individuals, two forensic psychiatrists (IO, ML), and a medical student (JR). Articles with differing opinions on inclusion/exclusion were discussed together until consensus was reached.

**Figure 1:**
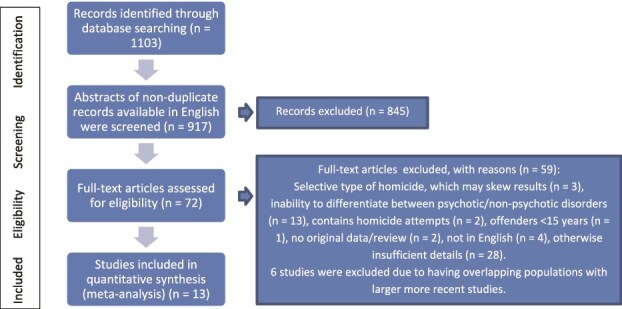
PRISMA Flowchart of the Study Process.

A total of 1103 articles were screened and 186 articles not available in English removed. Out of the 917 individual articles 845 were discarded through screening of abstracts. A total of 72 full-text articles were assessed for eligibility and 53 were discarded for reasons consistent with the exclusion criteria. The remaining 19 articles were analyzed for bias using the Newcastle-Ottawa Scale for assessing the quality of non-randomized studies in meta-analyses (https://www.ohri.ca/programs/clinical_epidemiology/oxford.asp). Data on the total number of homicide offenders as well as the number of victims previously unknown to the assailant were extracted from the articles for the meta-analysis. Six studies were found to overlap in their population. Of these overlapping studies, the larger studies were included and smaller studies excluded (the larger studies were later publications including the earlier datasets supplemented with data from longer follow-up). A total of 13 studies were included in the quantitative meta-analysis. This process and the PRISMA flow chart are displayed in Figure 1.

### Statistical Analysis

Data for total number of homicide offenders as well as the number of victims previously unknown to the assailant were extracted from the articles for the meta-analysis. A random-effects meta-analysis using meta and metafor packages in R version 4.4.2 was conducted using the restricted maximum-likelihood (REML) method to account for variability across studies. The primary outcome was the pooled rate of stranger homicides among individuals with a psychotic disorder who committed a homicide, expressed as a proportion with 95% confidence intervals (CIs). Heterogeneity was assessed using Cochran’s Q-test and I^2^ statistic. Potential publication bias was assessed using visual inspection of funnel plots, Egger’s regression test for funnel plot asymmetry, and the trim-and-fill method to estimate and adjust for missing studies. We also performed meta-regressions using age, publication year, and percentage of males to explore potential sources of heterogeneity.

A sensitivity analysis was conducted by leaving out the studies with greatest bias (receiving only one or two stars on the Newcastle-Ottawa scale).

### Ethical Approval

Ethical approval was not sought for this study, due to its registry-based nature.

## Results

Of the 917 articles screened, 13 were included in the meta-analysis. The studies and their data are shown in Table 1 and have been summarized below.

**Table 1:** List of Included Studies, Including Their Publication Year, Country Where Data Originates From, Data Extracted and Newcastle-Ottawa Scale Score for Analysis of Bias.

Author(s)	Pub. year	Psychotic homicide offenders	Total victims	Stranger victims	% of offenders having stranger victims	Country and years of the sample	Newcastle-Ottawa scale score (bias)
Balcioglu et al.	2024	121		9	7,4	Türkiye and Russia, 2012-2022	3
Nielssen et al.	2022	169	169	13	7,7	Australia, 1993-2016	3
Golenkov et al.	2021	179	200	7	3,9	Russia, 1981-2020	2
Ojansuu et al.	2022	389	414	40	10,3	Finland, 1980-2014	3
Hachtel et al.	2021	43		4	9,3	Australia, 1997-2005	3
Chen et al.	2018	107	>116	20	18,7	China, 1998-2006	1
Belli et al.	2010	49		6	12,2	Türkiye, 2004-2007	1
Meehan et al.	2006	85		12	14,1	UK, 1996-1999	2
Nordtsröm et al.	2003	52	56	9	17,3	Sweden, 1992-2000	2
Nestor et al.	1995	19	23	2	10,5	USA, 1987-1995	2
Vielma et al.	1993	58		11	19,0	UK, 1987	2
Gottlieb et al.	1987	58	66	2	3,4	Denmark, 1959-1983	3
Benezech et al.	1984	109	126	42	38,5	France, 1977-1981	3
Total		1438		177			

### Description of the Included Articles

Balcioglu et al.^28^ included a total of 121 individuals (92 from Türkiye (Turkey) and 29 from Russia) with schizophrenia who had committed 9 stranger homicides (6 in Türkiye, 3 in Russia) during the years 2012 and 2022 (stranger homicides 7.4% overall; 6.5% in Türkiye and 10.3% in Russia). Most of the sample was collected from a forensic center in Istanbul, Türkiye and a minority from a forensic center located in the Chuvashia region of the Russian Federation. The Turkish center served a population of 27 million and the Russian center a population of 1.4 million individuals. Russian subjects were more educated, had more lifetime suicide attempts, longer illness duration, higher rates of blunt traumatic homicides, higher rates of intoxication with alcohol or substances, and lower rates of experiencing delusions at the time of the index homicide compared to their Turkish counterparts.^28^

Balcioglu et al.^29^ had also published a prior study including 47 homicide perpetrators with schizophrenia who had committed 4 stranger homicides from the forensic center in Istanbul, Türkiye between the years 2018 and 2021 (8.5% stranger homicides). Forty-four (93.6%) of the homicide incidents were psychotically motivated.^29^ As the population in this study was likely included in the above-mentioned study,^28^ this study population was left out from the meta-analysis.

Nielssen et al.^30^ included 169 individuals found not guilty by reason of mental illness (NGMI) who had committed 13 stranger homicides between 1993 and 2016 in the New South Wales area of Australia (7.7% stranger homicides). Over this period, the rate of non-NGMI homicide convictions fell from 1.83 per 100,000 per annum to 0.65 per 100,000 per annum, while the rate of NGMI homicide fluctuated, with an average annual rate of about 0.1 per 100,000 per annum. There was no association between the annual rates of NGMI and non-NGMI homicides, but the falling rate of non-NGMI homicide meant that the proportion of NGMI offences doubled from 5.5% in the first 12 years to 11% in the second 12 years. Most (83.4%) of the NGMI offenders had previous contact with mental health services, but only half of these had received treatment with antipsychotic medication.^30^

Golenkov et al.^31^ included 179 individuals diagnosed with schizophrenia who committed 7 homicides with stranger victims during 1981 to 2020 in the Chuvashia region of the Russian Federation (3.9% were thus stranger homicides). Of the offenders 19 were female and did not have stranger victims. The mean age of the offenders was 36.8 years and 56.4% lived in a rural location. They report that the general annual homicide rate first rose from a rate of 9 per 100 000 in 1980s, peaked at 17 per 100 000 in the 2000s and then fell to 6 per 100 000 in the 2010s. The rate of homicides by individuals with schizophrenia had a similar trend, starting at 0.28 per 100 000 in the 1980s, peaking at 0.47 per 100 000 in the 2000s and then declining to 0.21 per 100 000 in the 2010s.^31^

Ojansuu et al.^32^ included 389 psychotic perpetrators responsible for 414 homicides, of which 40 were targeted at strangers. The cohort was collected from Finland between the years 1980 and 2014 from forensic psychiatric case files and included both male and female offenders. The article describes in detail the relationships between the victims and offenders. The study also included incidents of stranger homicides for both psychotic and non-psychotic perpetrators from nationwide databases.^32^

Hachtel et al.^13^ included 435 perpetrators of whom 43 were psychotic. They included in the psychotic group patients with schizophrenia, schizoaffective disorders, severe depressive episodes with psychotic symptoms or delusional disorder. These patients committed 43 homicides (including murder, manslaughter, infanticide and filicide) targeting 4 strangers, thus giving a stranger homicide rate of 9.3 %. Their sample was collected from cases between 1997 and 2005 using the police database and the psychiatric case register. They also analyzed the sex, motives, medications, and co-offender of the perpetrators. They found that female perpetrators were more likely to have a psychosis diagnosis than men. They found that the victims of the psychotic group were most often family members or other relatives. The psychotic offenders were more likely to act alone than other perpetrators, and their motivation was more often revenge. Hachtel et al. suggested that delusions and severed perception of reality, which are symptoms of psychotic disorders, might be mediators of the increased homicide rate among psychotic offenders.^13^

Chen et al.^33^ included only patients with schizophrenia in their study. They took a random sample from police and court records of 20% of the perpetrators with schizophrenia who committed a homicide between the years 1998 and 2006, ending up with 107 perpetrators with schizophrenia, nine of whom had multiple victims. Twenty of the perpetrators had targeted a stranger, yielding a stranger homicide rate of 18,7%. Overall, family and relatives were the most likely victims. Chen et al. also discovered that only about 40% of the perpetrators had ever been treated for psychosis and only four were undergoing treatment at the time of homicide. They also found that perpetrators with schizophrenia were less likely to be substance abusers but more likely to be older, less educated, at higher risk for recidivism, and to live in more rural areas than perpetrators without a diagnosis.^33^

Another study sample by Golenkov et al.^34^ was collected from an environment with a high overall homicide rate in Russia. They discovered 171 homicide cases with perpetrators diagnosed with schizophrenia during the years 1981 to 2010, but were only able to obtain records from 141 of these cases. These cases involved 133 individual perpetrators (120 males, 13 females;8 with two separate homicide cases) with a total of 155 victims, of whom 9 were strangers, yielding a stranger homicide rate of 6.8%. Nine of the homicides had multiple victims within a single incident, and those nine had a total of 23 victims. The mean age of the offenders was 34.8 years. They found that 58,9% of the perpetrators had psychotic symptoms (hallucinations, delusions) during the homicide and the rest (41%) were suffering from excessive impulsive behavior or lack of empathy. They also noted that their sample was less educated and had a worse social-economic situation than the general population of the area. One hundred and five of the offenders lived in a rural area or small towns, and three-quarters were unemployed. They observed their homicide rate among the sample to be higher than what was reported in other countries for patients with schizophrenia, but thought it likely resulted from the overall higher homicide rate in the environment.^34^ As this study population was likely included in the later article by Golenkov et al. in 2021,^31^ it was not included in the meta-analysis.

Nielssen et al. (2011)^35^ included in their study a sample from Australia, 1992 to 2008, of individuals with schizophrenia, who had been found not to be criminally responsible. Their sample consisted of 138 homicide perpetrators, of whom 19 had targeted strangers, yielding a 13,8% stranger homicide rate. They also studied perpetrators accused of attempted homicide (n=134, not included in the present analyses). They found that patients without prior treatment had a higher homicide rate, but were less likely to target strangers than patients who had received treatment. They also noted that 97% of the perpetrators of either successful or attempted homicides were delusional or hallucinating during their attack. This percentage, significantly higher than in some other studies, might reflect their sampling of only offenders deemed not legally responsible.^35^ As this study population is likely included in the later article by Nielssen et al. in 2022,^30^ it was not included in the meta-analysis.

Belli et al.^36^ collected their sample from Türkiye between the years 2004 and 2007. Their sample included 43 men and 6 women (mean age 37.0 years), all of whom suffered from schizophrenia, had committed homicide, and were in a forensic hospital under compulsory treatment at the time of sampling. The 49 offenders had a total of six stranger victims, yielding a stranger homicide rate of 12,2%. Interestingly, only 20 % of the sample (0% of the females) had used their prescribed antipsychotic medications during the offense.^36^

Nielssen et al. (2007)^37^ studied homicides in Australia during the years 1993-2002. They included in their study individuals with any form of psychotic illness (schizophrenia or other disease with psychotic symptoms). They collected their sample from court, police, medical and other official records (mental health tribunal). They detected 88 perpetrators (including 17 females; mean age of total sample 33.4 years), who had five stranger victims, yielding a stranger homicide rate of 5.7%. A majority (61%) of the perpetrators were first episode patients and 57% had reported feeling threatened prior to their assault.^37^ As this study population is likely included in the later article by Nielssen et al. in 2022,^30^ it was not included in the meta-analysis.

Laajasalo and Häkkänen (2006)^38^ collected their data from Finnish police and forensic mental state examination registers. They found 125 individuals (12 female; mean age 45,7 years) with schizophrenia who had committed a homicide in Finland between the years 1992 and 2004. The 125 offenders had a total of 134 victims of whom 10 were strangers, yielding a stranger homicide rate of 8%. There were 8 cases with multiple victims and 12 co-offending cases in their sample. 92.8% of the offenders were delusional or hallucinating during their offense, and 66.4% of the offenses had a psychotic motive.^38^ As this study population is likely included in the later article by Ojansuu et al. in 2022,^32^ it was not included in the meta-analysis.

Meehan et al.^9^ had a sample of 1594 homicides with 85 perpetrators with schizophrenia or other delusional disorder who had committed homicide in England or Wales between the years 1996 and 1999. Twelve of them had killed a stranger, yielding a stranger homicide rate of 14.1%. They collected their data from police, court, health service, and other official registers. 67% of the perpetrators had delusions, and 56 (66 %) were first episode patients.^9^

Nordström et al. (2006)^39^ collected all homicides by patients who had a schizophrenia diagnosis between 1992 and 2000 in Sweden. They found 48 male offenders who committed 52 homicides and 9 stranger victims. The mean age was 32.3 (19-58). They also found 4 female offenders who committed 4 homicides, but none of the victims were strangers to the offender. Given this, the total stranger homicide rate was 17.3%. They compared two groups of homicides; family victim -group (FV-group) which included siblings, parents and grandparents and other victims –group (OV-group). They found that FV-group had more hallucination/delusions than OV-group and 81% of the FV-group homicides took place at the victim’s home. OV-group had more intoxication for both offender and victim than OV-group. They also found that only 33% of the offenders were under psychiatric treatment and only 4.2% had used medication at the time of homicide.^39^

Nestor et al. (1995)^40^ included data from forensic hospitals’ patient folders from Massachusetts between the years 1987 and 1995. They found 19 male patients with schizophrenia or other disorders with psychotic symptoms, who had committed a total of 23 homicides with two stranger victims, yielding a stranger homicide rate of 10.5%. The mean age of the patients was 32,5 years. They also performed a more in-depth analysis of the delusions suffered by the offenders and how they were connected with the violence.^40^

Nestor & Haycock (1997)^41^ may have partially overlapping data with Nestor (1995).^36^ They examined “not guilty by reason of insanity”-patients from a forensic hospital in Massachusetts between the years 1987 and 1991. They detected 13 male patients (mean age 35.2 years) who had committed a total of 17 homicides with only one stranger victim, yielding a stranger homicide rate of 7.7%. Twelve of the thirteen patients had psychotic symptoms during their offense.^41^ As this study population is likely included in the prior article by Nestor et al. in 1995,^40^ it was not included in the meta-analysis.

Vielma et al. (1993)^42^ studied male patients from a single hospital in 1988 in England. They collected their data with a questionnaire given to patients and supplemented the information from the staff of the hospital when information was missing. They found 58 psychotic offenders who killed a total of 11 strangers. Stranger homicide rate was thus 18.9%. The mean age of the males was 31. Only 25% of them were under psychiatric treatment and over three-quarters of them were unemployed. Stabbing was the most frequent method used to commit homicide.^42^

Gottlieb et al.^43^ studied every homicide offender who had been under a psychiatric examination in Copenhagen, Denmark, between the years 1959 and 1983. Their data came from psychiatrists’ statements given to the courts. They detected 58 psychotic perpetrators (16 females; mean age 33 years), who had a total of 66 victims of whom 2 were strangers, yielding a stranger homicide rate of 3.4%.^43^

Benezech (1984)^14^ studied psychotic patients involved in homicide and treated in state hospitals between 1977 and 1981 in France. They found that there were 101 psychotic male patients and 8 females meeting the criteria. These patients committed a total of 126 homicides, which included 42 stranger victims, yielding a stranger homicide rate of 38.5 %. Patients with schizophrenia (n=64) were responsible for 79 victims, including 29 strangers, and the rest of the acts were committed by patients with a diagnosis of paranoia, bipolar disorder or other psychosis.^14^

### Pooled Rate of Stranger Homicides

**Figure 2:**
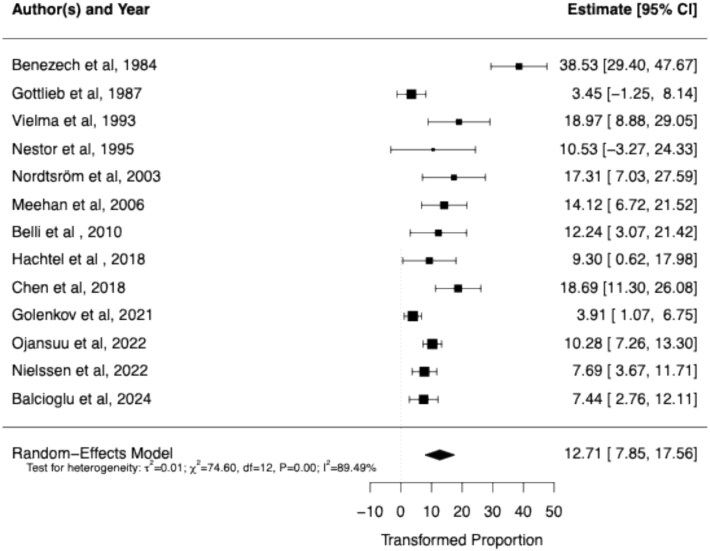
Pooled Rate of Stranger Homicides.

**Figure 3:**
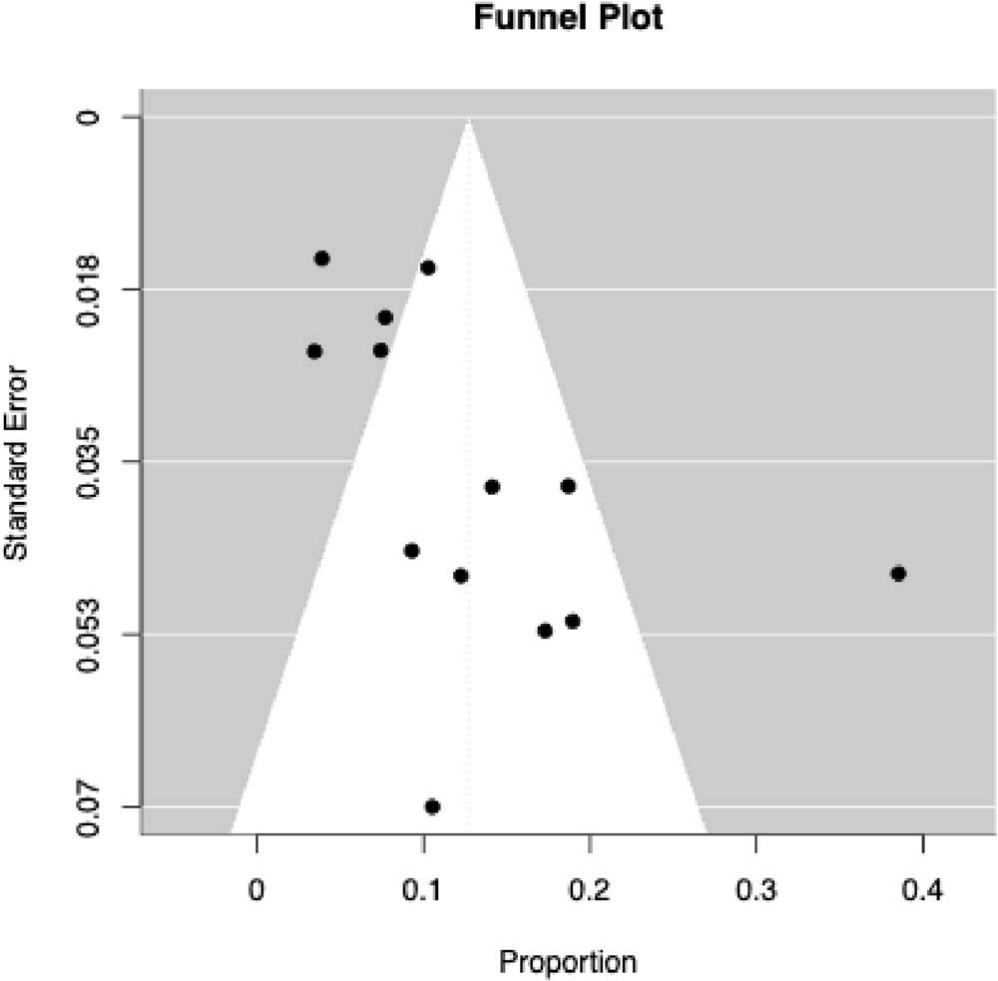
Funnel Plot of Studies Included in Meta-Analysis.

The pooled rate of stranger homicides among individuals with a psychotic disorder was 12.7% (95% CI: [7.85, 17.56]). These results are shown in Figure 2. The z-test for the pooled estimate was highly significant (Z=5.13, P<0.0001). Substantial heterogeneity was observed among the included studies (I^2^=89.49%, Q=74.60, df=12, p<0.0001). Meta-regression analyses did not reveal any significant differences in stranger homicide proportions by publication year or average age proportion (all p-values>0.05). However, we found that the proportion of males explained 26.9% of between-study variance (P-value<0.05). Visual inspection of the funnel plot suggested asymmetry and Egger’s regression test confirmed significant funnel plot asymmetry (Z=2.22, P-value=0.03, Figure 3). However, the trim-and-fill method identified zero potentially missing studies.

Four studies also included data on gender distributions, but as the number of studies was rather low, no meta-analysis was conducted by gender.

## Discussion

The results of this meta-analysis indicate that about 13% of psychotic homicide offenders target strangers. Although the number is slightly higher than that (9 %) published in a previous meta-analysis by Nielssen et al.,^16^ it still indicates that most of the homicides by psychotic individuals are targeted at individuals previously known to the offender, most often family members or relatives. It is important to note that in general, being the victim of a homicide committed by a psychotic individual is extremely rare. For example, in Finland the incidence of stranger homicide committed by individuals with psychosis has been reported to be 0.022 per 100 000 person-years.^32^

Only four of the studies included in the previous meta-analysis^16^ met our inclusion criteria. Out of these four studies, the datasets of two studies have been included in more recent publications with longer follow-up times and broader data. Thus, only two of the articles that were included in the previous meta-analysis were included in the meta-analysis presented here. In addition to those, eleven other studies were identified (including the two with broader datasets). Seven of these have been published after the initial meta-analysis^16^ and four prior to that. In their previous meta-analysis Nielssen et al. used a fixed-effects model, but as we estimated the heterogeneity of the dataset to be very large, we opted to use a random effects model. It should also be noted that their study included articles with homicide attempts, which we excluded. These are likely, but may not be all-encompassing, explanations for the differences in the stranger homicide rate observed between these meta-analyses.

Families and friends are an important support for people with schizophrenia and facilitate the recovery of the patient.^44^ However, family members of people with a psychotic disorder are at higher risk of becoming victims than others.^45^ Thus, families of individuals with psychotic disorder need early support for their own well-being^44^ and to reduce the risk of violence within the family.^45^ Also, of note, is that a study found that individuals suffering from schizophrenia and committing a homicide are at higher odds of having another individual with schizophrenia in the family (38,8%), indicating that the risk factors for committing homicide and having schizophrenia may be similar.^36^

Interestingly, many psychotic homicide offenders were found to be experiencing a first psychotic episode. A large majority of the offenders in general committed their crimes while suffering from delusions or psychotic symptoms. Thus, early discovery of these disorders and their decisive treatment would likely help to reduce the number of homicides committed by psychotic individuals. Although these homicides are tragic events for the victims and their families, they are also often traumatizing for the patients committing them, as they are often committed due to psychotic motives, bewildering and frightening for the perpetrator who has recovered from his/her psychotic episode.

During the literature review process for the meta-analysis we also found interesting articles pertaining to the possible risk factors behind targeting strangers, although factors related to victim selection have still been largely understudied. It has been shown in a study by Laajasalo and Häkkänen that excessively violent homicides committed by psychotic individuals are less likely to have stranger victims (2.7 %) than homicides without excessive violence (9.4 %).^38^ Also, the risk of targeting a stranger has been shown to be higher on later psychotic episodes (32 %) as opposed to the first episode (28 %).^35^ Some studies have shown stranger homicides by psychotic individuals to be more common in rural areas (6.8 % vs. 18.7 %), but as these studies are scarce and the phenomenon has not been systematically studied in different countries, it is still under debate.^34^ Rural societies may have less developed justice systems, and it might not take a great leap to assume that societies with less developed justice systems might more easily convict psychotic individuals of crimes where evidence is sparse, but pressure for convictions is high, as these individuals may be less able to fend for themselves.

Although the stranger homicide risk of psychotic individuals was shown to be higher than previously reported, it should be especially noted that the rate of stranger homicide is still higher among non-psychotic antisocial individuals than psychotic offenders,^6^^,^^7^^,^^32^ although homicides committed by psychotic individuals and targeted at strangers often result in far wider media coverage and result in greater public outrage. This is alarming as it might increase the already marked stigma patients with psychotic disorders suffer from.

While there is no known effective pharmacological treatment for antisocial personality disorder or psychopathy,^46-48^ which may be comorbid with psychotic disorders, other risk factors for homicides and violence observed in individuals suffering from psychotic disorders can often be treated and the risk of violence in these individuals reduced.^49^

The strengths of this study are a systematic literature review completed by three independent individuals, including two specialists of forensic psychiatry, prior to meta-analysis of the results. The power of this study is also greater than the earlier meta-analysis due to a larger set of source material.

There are also some limitations to this study. As data were harvested from publications with often incompletely reported information some misclassifications can occur. There is also a risk that some of the articles have remaining overlapping data, even though we tried to exclude clearly overlapping studies. Homicide is a rare phenomenon in general, and even rarer is the homicide committed by an individual with a psychotic disorder and targeted at a stranger. Therefore, single cases can have a large impact on the overall dataset. Also, careful selection of studies is crucial, as bias is easily introduced with selection. For example, including a study on homicides committed by pushing people onto subway tracks, where all of the victims were strangers,^50^ would have been likely to skew the results markedly. One major issue is the definition of a stranger, which often varies between publications. Some publications might classify acquaintances as strangers, others only people not known for more than 24 hours and yet others only people that the assailant has never met before. The classification used, which unfortunately was not reported in all papers, may also affect results, likely leading to an overestimation of the proportion of stranger homicides, as people that are somewhat known to the assailant may be classified as strangers. Another weakness is the high heterogeneity of the studies. Pooling large studies from different parts of the world and spanning several decades has a high risk of introducing heterogeneity. Although we used meta-regression in an attempt to elucidate the sources of heterogeneity between the studies, we were only able to explain a part of it, which seemed to be due to variability in the proportions of sexes included in the articles. Other sources of heterogeneity might include the societal and cultural aspects of the country where the study samples were collected from (for example, availability of alcohol and substances of abuse, the healthcare system, the psychiatric treatment system, the judiciary system), the urbanicity of the study samples, the years of data collection and other factors that we were not able to extract reliably enough to be included in the meta-regression. As a result, the final result of the meta-analysis is an average that may not be generalizable locally. As an assessment of general risk on population levels, the results from this study should not be used to evaluate individual risk.

## Conclusion

Psychotic symptoms that drive violence are quite universal (persecutory delusions, misperceived threat, threat-control override symptoms, and disorganized thinking) and are probably biologically rather than culturally or societally driven. These symptoms may influence the overall propensity for violence more than they influence target selection. This can lead to convergent victim patterns across countries and cultures, as the targets may simply be the people who happen to be closest to the individual experiencing symptoms. It is also a fairly universal phenomenon that family members and friends are the ones most likely to support the patient when the first symptoms of psychosis emerge, which increases their likelihood of becoming victims of violence or homicide. Consequently, homicides involving stranger victims tend to remain similarly rare events across different countries and cultures.

In general, being the victim of a homicide committed by a psychotic individual is extremely rare. However, our meta-analysis shows that the proportion of homicides targeted at strangers committed by psychotic individuals is higher than has previously been reported. While there is no known effective treatment for antisocial personality disorder or psychopathy, other major causes for homicides and violence, psychotic disorders can often be treated and the risk of violence among individuals suffering from these disorders markedly reduced. As most of the homicides committed by psychotic individuals are driven by symptoms of their disorder they could potentially be prevented with effective treatment. Also, more research is needed on the specific risk factors leading to homicides targeted at strangers to prevent these tragic events. Such risk factors could include: living standard, specific symptoms, perpetrator age or features such as sex or age of the victim. However, as a homicide in itself is a tragic event, efforts should be concentrated on reducing the number of homicides committed by psychotic individuals, regardless of whether the victim is a stranger or not.
